# Unintended Presence of Human Cilium in the Anterior Chamber After Phacoemulsification: A Report of Two Cases and Literature Review

**DOI:** 10.7759/cureus.80360

**Published:** 2025-03-10

**Authors:** Kayvon A Moin, Amin Ashrafzadeh, Paul Phillips, Phillip C Hoopes, Majid Moshirfar

**Affiliations:** 1 Ophthalmology, Hoopes Vision Research Center, Draper, USA; 2 Ophthalmology, Nassau University Medical Center, East Meadow, USA; 3 Ophthalmology, Modesto Eye Center, Modesto, USA; 4 Ophthalmology, Sightline Ophthalmic Associates, Wexford, USA; 5 John A. Moran Eye Center, University of Utah School of Medicine, Salt Lake City, USA; 6 Eye Banking and Corneal Transplantation, Utah Lions Eye Bank, Murray, USA

**Keywords:** anterior chamber, cataract surgery, cilia, cornea, crystalline lens, eyelash, intraocular foreign body, intraocular lens, ophthalmology, sutureless corneal wound

## Abstract

Cataract surgery has consistently demonstrated safety and efficacy in restoring vision through the removal of visually-significant cataracts. Advances in surgical techniques and technology have led to excellent visual outcomes and minimal complications. Despite this, rare complications can occur, such as the introduction of intraocular foreign bodies, including cilia. Although extremely uncommon, the presence of cilia in the eye post intraocular surgery poses potential risks, including infection and inflammation. This study presents two distinct cases of intraocular cilia found in the anterior chamber following crystalline lens extraction with phacoemulsification. In both cases, cilia were identified shortly after the procedures, despite any signs of foreign bodies at the conclusion of the surgical procedure. This study also explores potential mechanisms for cilia migration into the eye, including the self-sealing nature of clear corneal incisions and the characteristics of cilia through a brief literature search of reported cases in the current literature. This study highlights the need for careful surgical practices to minimize the risk of foreign body complications and underscores the importance of continued research into intraocular foreign body management.

## Introduction

Modern phacoemulsification has been a safe and effective procedure for the removal of visually significant cataracts for several decades. With advancements in surgical techniques and technology, patients have been able to achieve excellent visual outcomes with minimal risk of complications [1]. Foreign bodies such as metals from prolonged instrument usage, lint fibers from surgical wipes and gauzes, plastic from instrument wrapping, and intraocular lens defects have been reported [2,3]. While it is assumed that these foreign bodies are most often introduced during surgery, a rare but potentially serious complication that could arise is the introduction of an intraocular foreign body after surgery. One rare example of an intraocular foreign body after cataract surgery is a cilium (eyelash), with only a handful of cases reported globally [4-12]. Some studies have attempted to rationalize the mechanism by which cilia migrate intraocularly, considering the self-sealing nature of clear corneal incisions used in cataract surgery and the absence of any clinical signs of foreign bodies in the immediate conclusion of the surgery. Although seemingly benign, the presence of cilia inside the eye may expose the patient to potential pathogens, putting them at risk for developing endophthalmitis [6]. 

In this study, we present two cases from two different surgeons in which cilia were found in the anterior chamber of the eye shortly after phacoemulsification crystalline lens extraction, despite the absence of any signs of their presence at the conclusion of the procedure.

## Case presentation

Case 1

A 68-year-old highly myopic astigmatic male (left eye {OS}: -12.00 -2.50 x164) without any pertinent past medical history underwent cataract extraction with phacoemulsification and toric intraocular lens (IOL) insertion in the left eye. With informed consent, the procedure was performed under topical anesthesia. Prior to starting the procedure, the eye and adnexa were sterilized with 5% povidone-iodine and the patient was properly draped. The eyelashes were taped with Tegaderm without any eyelash trimming. A 2.4 mm temporal clear corneal incision was made with a keratome. A 5.5 mm continuous curvilinear capsulorhexis was made with a cystotome followed by hydrodissection and hydrodelineation with an irrigation cannula. Nuclear and cortical lens materials were removed with phacoemulsification and irrigation/aspiration handpieces, respectively. A cohesive viscoelastic agent was injected into the capsular bag and a one-piece +8.5 D Clareon Toric IOL (CCW0T4; Fort Worth, TX: Alcon Laboratories) was inserted. Any residual viscoelastic material was removed. Intracameral moxifloxacin 0.16% ophthalmic solution was administered at the conclusion of the procedure. The temporal clear corneal incision was adequately hydrated for complete wound closure. No foreign bodies were observed intraocularly or on the ocular surface. A fox shield was placed over the left eye. The surgery was uneventful, and the patient was instructed to use routine ofloxacin 0.3% ophthalmic solution four times daily for one week, ketorolac tromethamine 0.5% ophthalmic solution four times daily for two weeks, and prednisolone acetate 1% ophthalmic suspension drops four times daily, tapered weekly over one month following surgery.

**Figure 1:**
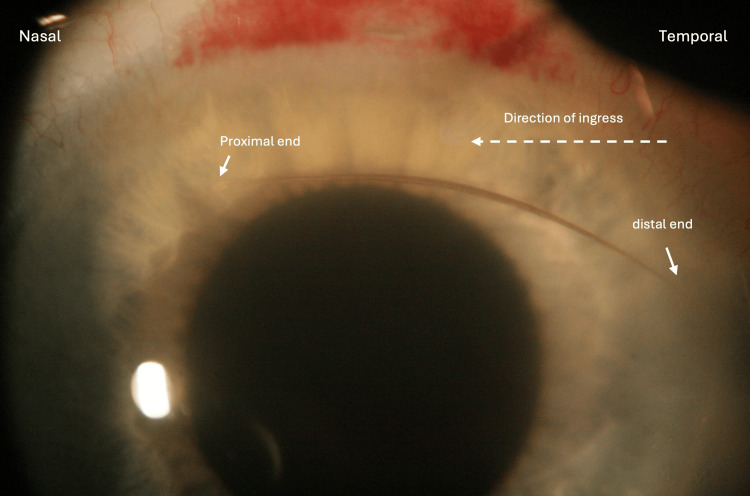
Cilium in the anterior chamber (AC) one day after phacoemulsification cataract extraction of the left eye.

On postoperative day one, the patient reported improvement in vision with mild halo symptoms in the left eye but denied any pain or discomfort. Uncorrected distance visual acuity (UDVA) was 20/25 and intraocular pressure (IOP) was 18 mmHg in the left eye. Slit lamp examination (SLE) of the left eye demonstrated mild corneal wound edema, 1+ cells, and an 8-10 mm cilium in the anterior chamber (AC), with the proximal end oriented nasally and the distal end oriented temporally (Figure 1). The posterior chamber IOL (PCIOL) was centered and was in a good position. The Seidel test was negative. After discussion and informed consent, the patient returned to the operating room the same day and the left eye was prepped and draped in a sterile fashion. A grasping 25 gauge MST intraocular forceps (Redmond, WA: MicroSurgical Technology) was used to remove the cilium from the AC through the same temporal clear corneal incision used for cataract extraction. Preservative-free moxifloxacin 0.5% ophthalmic solution was injected into the AC and the temporal corneal wound was then hydrated and meticulously checked for proper closure. The patient returned the next day and was observed to have no complaints or significant findings on SLE. The remainder of the postoperative follow-up proceeded as expected.

Case 2

A 51-year-old hyperopic female (right eye {OD}: +4.25 -1.50 x012; OS: +4.00 -1.00 x158) with a past ocular history of hyperopic laser-assisted in situ keratomileusis (LASIK) in both eyes (OU) 26 years prior and bilateral upper and lower lid blepharoplasty six years prior underwent clear lens exchange (CLE) with +28.0 D light adjustable lenses (LAL+) (Aliso Viejo, CA: RxSight) in both eyes simultaneously for correction of progressively blurry distance vision. Her medical history includes depression controlled with citalopram, escitalopram, lorazepam, and paroxetine. After informed consent the procedure was performed under topical anesthesia, and the patient was properly draped, and eyelashes were taped without eyelash trimming. The surgical technique was identical to the previously described case, with the exception of expanding the 2.4 mm temporal clear corneal wound to 3.0 mm OU for IOL insertion. No foreign bodies or cilia were noted intraocularly or on the ocular surface at the end of the procedure. A pair of protective (ultraviolet {UV}-blocking) sunglasses were placed over the eyes. The patient was instructed to use prednisolone 1%, moxifloxacin 0.5%, and bromfenac 0.075% eye drops postoperatively.

**Figure 2:**
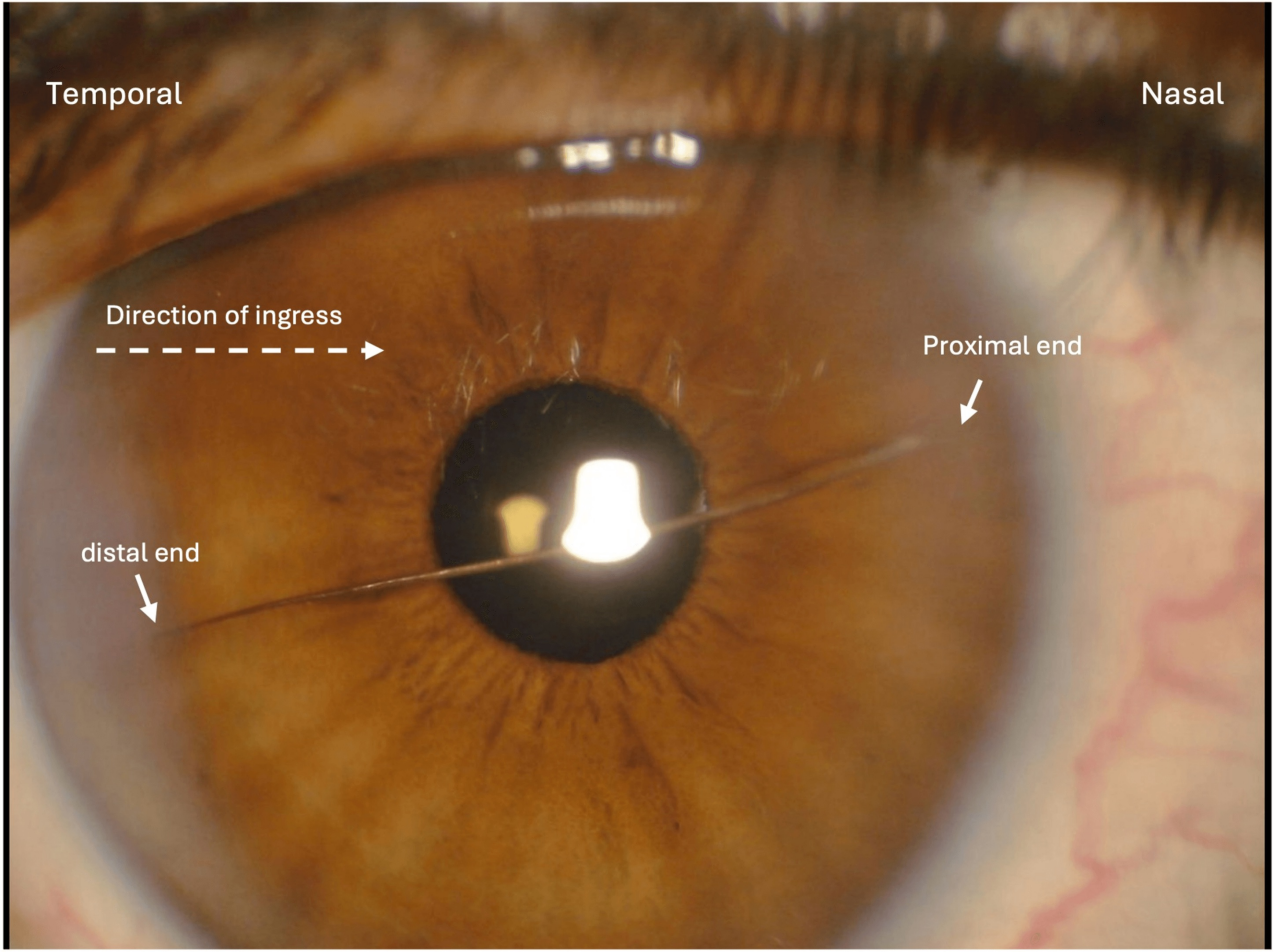
Cilium in the anterior chamber (AC) one day after phacoemulsification clear lens exchange (CLE) of the right eye.

On postoperative day one, a large, horizontally oriented cilium was observed in the anterior chamber of the right eye, with the distal tip incarcerated at the temporal corneal incision site and the proximal end oriented nasally (Figure 2). Minimal cells/flare were present. The Seidel test was negative. The PCIOLs in both eyes were centered and in a good position. The incarcerated distal tip of the cilium was grasped with the Jeweler’s forceps and removed at the slit lamp, with the AC remaining fully formed. Moxifloxacin 0.5% ophthalmic solution was applied topically post removal and the patient was instructed to continue routine postoperative regimen. The patient returned two days later for a follow-up visit. There was no presence of cilia intraocularly, but there were rare cells present in the AC in both eyes. The patient was instructed to continue the moxifloxacin drops and continue prednisolone and bromfenac. At the time of writing, the patient is more than six months post-surgery and has had no signs of intraocular inflammation or infection. 

## Discussion

As mentioned previously, intraocular cilia after phacoemulsification crystalline lens extraction is a very rare finding, with only nine other cases reported globally (Table 1). Across these studies, nine cases were reported with presentation time varying from one day to 16 years postoperatively [5]. Similar to both of our cases, 22% of reported cases (two out of nine) in the current literature presented with intraocular cilia at postoperative day one. Given that over 26 million cataract surgeries are performed annually across the world, the estimated incidence rate is well under one in one million cases per year, although this number is likely highly limited by hesitations to report complications of this nature [13].

**Table 1:** Overview of all reported cases of intraocular cilia after phacoemulsification crystalline lens extraction. M: male; F: female; AC: anterior chamber; PC: posterior chamber; IOL: intraocular lens; PKP: penetrating keratoplasty

Author	Age, sex	Time of presentation	Intraocular location	Orientation	Corneal wound site	Clinical findings	Management
Humayun et al. (1993) [5]	61, F	16 years	AC (embedded in iris)	Vertical	-	Corneal decompensation; no intraocular inflammation	PKP; cilium removed
Galloway et al. (2004) [6]	81, M	1 week	AC	Horizontal	Temporal	Anterior uveitis and endophthalmitis; negative vitreal sampling	Intravitreal antibiotics and steroids; cilium removed
Islam and Dabbagh (2006) [7]	79, M	3 months	AC	Oblique	-	Asymptomatic; no intraocular inflammation/infection	Observation
Rofail et al. (2006) [8]	60, M	3 days	AC, with migration into the PC	Vertical	Temporal	Ocular irritation; no signs of intraocular inflammation/infection	Observation
Walker et al. (2007) [9]	75, M	6 weeks	Embedded in main corneal wound site	Vertical	Temporal	Asymptomatic; no signs of intraocular inflammation/infection	Cilium removed
Etter and Kim (2008) [10]	79, F	1 day	Embedded in main corneal wound site	Horizontal	Temporal	Minimal AC inflammation	Cilium removed
Bach et al. (2015) [11]	75, F	5 years	Posterior to IOL inside the capsule	Oblique	-	Asymptomatic; no signs of intraocular inflammation/infection	Observation
Stanley (2020) [12]	69, F	1 day	AC	Horizontal	Temporal	Minimal AC inflammation	Cilium removed
Cheong and Wee (2022) [4]	69, M	1 week	AC	Vertical	Temporal	Minimal AC inflammation	Cilium removed
Current study	68, M	1 day	AC	Horizontal	Temporal	Minimal AC inflammation	Cilium removed
	51, F	1 day	AC	Horizontal	Temporal	Minimal AC inflammation	Cilium removed

The mechanism by which cilia can migrate intraocularly despite adequate corneal wound hydration and closure is the key concern. Taban et al. investigated the ingress of ocular surface fluid into cadaveric globes with sutureless clear corneal wounds using India ink. Their study revealed a significant increase in spectrophotometric readings in globes with sutureless wounds compared to those with sutured wounds. This increase correlated with microscopic observations of India ink particles in the anterior chamber, indicating fluid entry through the unsutured wounds [14]. All of the reported cases, including our own, utilized a sutureless technique for clear corneal wound closure, thus potentially explaining how cilia, typically with a width of 205±28 µm, may enter the eye [15]. Additionally, the majority of observed cases were performed through a temporal clear corneal incision, thus implicating the association between intraocular cilia migration and wound location.

Others have also theorized that the cellular structure of cilia may contribute to its migration and orientation within the eye. An electron microscopy analysis of the retrieved intraocular cilium by Rofail et al. demonstrated that the cuticle layer, the outermost layer of the cilium, consisted of overlapping cells arranged like shingles on a roof, with the free margin pointing toward the distal (tip) end. This supports the notion that the cilium may only pass through tissue with the proximal (follicle) end first [8]. Weiand et al. further explained that frictional forces on hair are anisotropic due to the overlapping structure of the outer cuticle cells. Specifically, friction is much higher when the hair is rubbed in the tip-to-root direction, as the cuticles interlock more tightly. In contrast, friction is lower when the hair is rubbed in the root-to-tip direction, allowing the cuticles to slide more freely over one another [16]. This observation provides additional support for the argument that the follicle or root end should enter the anterior chamber first. Others have also reported on the orientation of cilia once enter the eye. In 44% of the reported cases (four out of nine) in the current literature, cilia were observed with a vertical orientation [4,5,8,9], whereas the remaining 56% (five out of nine) were either horizontal or oblique [6,7,10,11,12]. Given the scarcity of reported cases, however, any conclusions regarding the orientation of cilia in relation to its intraocular migration remain speculative.

As with any intraocular foreign body, inflammation and infection are important secondary complications to consider. Galloway et al. reported a patient who developed fibrinous uveitis and exogenous endophthalmitis three days after cataract surgery due to an intraocular cilium. Microbial examination via vitreal sampling was negative and the cilium was not cultured [6]. Fortunately, the cilia in our cases were found and removed promptly before inciting any signs of infection. The other reported cases of intraocular cilia did not show any infection, despite some cases presenting up to 16 years later [5]. It is then reasonable to assume if the initial bacterial load is insufficient to induce inflammation or infection, then the cilia will most likely remain inert in the AC [6]. However, another potential complication to consider with intraocular cilia in the AC is endothelial cell loss leading to corneal decompensation, which may occur at any point in time after discovery [5]. Therefore, management of an intraocular cilium largely depends on not only the time of presentation, but the location of the cilium, any contact with the corneal endothelium, and the extent of ocular inflammation [5-7].

## Conclusions

To avoid this rare complication after cataract surgery, surgeons should maintain caution by properly wearing surgical caps, masks, and/or beard covers during surgery. All eyelashes should be covered adequately with the surgical drape and taped accordingly. If any foreign body is seen during the procedure, it should be flushed away immediately. After the procedure is finished, patients should be instructed to keep their eyes closed after the speculum is removed and to avoid blinking to prevent any fluid ingress or unintended protrusion of a cilium into the corneal wound. Both surgeons in our cases followed these recommendations and meticulously checked for both the presence of foreign bodies and complete corneal wound closure during surgery. This emphasizes the need for continued research into the mechanisms by which cilia may enter the eye, the impact of different surgical techniques, and the long-term implications of their presence.
